# The Frog *Xenopus* as a Model to Study Joubert Syndrome: The Case of a Human Patient With Compound Heterozygous Variants in *PIBF1*

**DOI:** 10.3389/fphys.2019.00134

**Published:** 2019-02-25

**Authors:** Tim Ott, Lilian Kaufmann, Martin Granzow, Katrin Hinderhofer, Claus R. Bartram, Susanne Theiß, Angelika Seitz, Nagarajan Paramasivam, Angela Schulz, Ute Moog, Martin Blum, Christina M. Evers

**Affiliations:** ^1^Institute of Zoology, University of Hohenheim, Stuttgart, Germany; ^2^Institute of Human Genetics, Heidelberg University, Heidelberg, Germany; ^3^Department of Neuroradiology, University Hospital Heidelberg, Heidelberg, Germany; ^4^Medical Faculty Heidelberg, Heidelberg University, Heidelberg, Germany; ^5^Division of Theoretical Bioinformatics, German Cancer Research Center (DKFZ), Heidelberg, Germany; ^6^Genomics & Proteomics Core Facility, German Cancer Research Center (DKFZ), Heidelberg, Germany

**Keywords:** PIBF1, Joubert syndrome, *Xenopus*, molar tooth sign, cilia, ciliopathy

## Abstract

Joubert syndrome (JS) is a congenital autosomal-recessive or—in rare cases–X-linked inherited disease. The diagnostic hallmark of the so-called molar tooth sign describes the morphological manifestation of the mid- and hind-brain in axial brain scans. Affected individuals show delayed development, intellectual disability, ataxia, hyperpnea, sleep apnea, abnormal eye, and tongue movements as well as hypotonia. At the cellular level, JS is associated with the compromised biogenesis of sensory cilia, which identifies JS as a member of the large group of ciliopathies. Here we report on the identification of novel compound heterozygous variants (p.Y503C and p.Q485^*^) in the centrosomal gene *PIBF1* in a patient with JS via trio whole exome sequencing. We have studied the underlying disease mechanism in the frog *Xenopus*, which offers fast assessment of cilia functions in a number of embryological contexts. Morpholino oligomer (MO) mediated knockdown of the orthologous *Xenopus pibf1* gene resulted in defective mucociliary clearance in the larval epidermis, due to reduced cilia numbers and motility on multiciliated cells. To functionally assess patient alleles, mutations were analyzed in the larval skin: the p.Q485^*^ nonsense mutation resulted in a disturbed localization of PIBF1 to the ciliary base. This mutant failed to rescue the ciliation phenotype following knockdown of endogenous *pibf1*. In contrast, the missense variant p.Y503C resulted in attenuated rescue capacity compared to the wild type allele. Based on these results, we conclude that in the case of this patient, JS is the result of a pathogenic combination of an amorphic and a hypomorphic *PIBF1* allele. Our study underscores the versatility of the *Xenopus* model to study ciliopathies such as JS in a rapid and cost-effective manner, which should render this animal model attractive for future studies of human ciliopathies.

## Introduction

**Figure 1:**
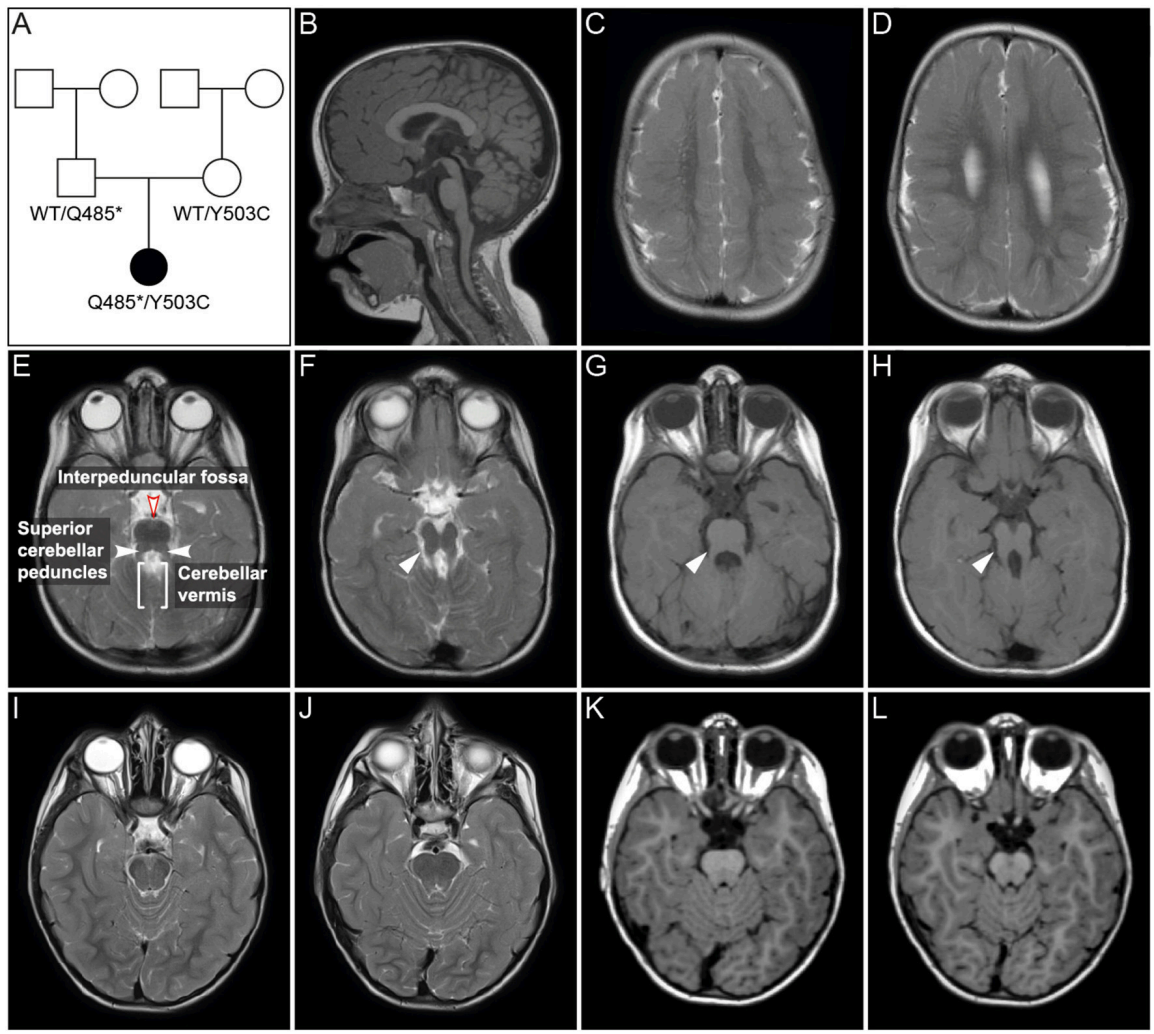
Pedigree and MRI scans. **(A)** Pedigree of patient family. Black symbol, affected individual; White symbols, unaffected individuals. **(B–H)** MR imaging of the patient at age 2 years and 6 months. Sagittal **(B)** and axial **(C–H)** images showed polymicrogyria in the parietal and temporal region **(C,D)** and hypoplasia of vermis cerebellum **(B,E–H)**. Axial MR images of cerebellum and brainstem **(E–H)** showed a mild “molar tooth sign” (marked with white arrows in **F–H**) due to a deep interpeduncular fossa, prominent and elongated superior cerebellar peduncles and a hypoplastic cerebellar vermis. **(I–L)** Corresponding MR images of a healthy control individual.

Joubert syndrome (JS, OMIM # 213300) comprises a group of autosomal recessive or X-linked inherited disorders with a distinct cerebellar and brainstem malformation recognizable on brain imaging, the “molar tooth sign.” The typical brain malformation of JS patients gives their midbrain an appearance reminiscent of a molar or wisdom tooth on axial MRI (Figures 1E–H). The “molar tooth” appearance results from three anatomical abnormalities of brainstem and cerebellum: (a) an abnormally deep “interpeduncular fossa,” (b) prominent, thickened, and elongated “superior cerebellar peduncles,” and (c) absence or hypoplasia of the midline portion of the cerebellum, the “cerebellar vermis” (see Figures 1E–H for the “molar tooth sign” and Figures 1I–L showing the corresponding MRI of a healthy control individual) (Maria et al., 1997, 1999). Typical clinical symptoms of JS are hypotonia, global developmental delay, intellectual disability, abnormal breathing pattern, abnormal eye movements, and cerebellar ataxia. Additional features include retinal dystrophy, cystic kidney disease, liver fibrosis, polydactyly, cleft palate, and facial dysmorphism in some patients (for review see (Parisi and Glass, 1993)). The estimated birth prevalence of JS is 1:80,000–1:100,000 (Parisi and Glass, 1993), but this may represent an underestimate due to many undiagnosed cases. A higher prevalence is found in the French–Canadian population, with several founder variants noted (Badhwar et al., 2000; Srour et al., 2012a,b, 2015). Founder variants in different genes have also been identified in the Canadian Hutterite, the Ashkenazi Jewish, and the Dutch population (Edvardson et al., 2010; Valente et al., 2010; Huang et al., 2011; Shaheen et al., 2014; Kroes et al., 2016). To date, pathogenic variants in more than 30 genes are known to cause JS (for review see Parisi and Glass, 1993). The encoded proteins of all these genes localize either to the primary cilium, basal body and/or centrosome and play a role in the formation, morphology, and/or function of these organelles, rendering JS a member of the rapidly expanding family of ciliopathies (Parisi and Glass, 1993; Romani et al., 2013). Common features of many ciliopathies include brain malformation, renal disease, retinal dystrophy, and polydactyly. Pathogenic variants in genes that cause Joubert syndrome have also been identified in ciliopathies with clinical findings that overlap with JS, e.g., Meckel-Gruber syndrome (MKS), Jeune asphyxiating thoracic dystrophy (JATD), Bardet-Biedl syndrome (BBS), oral-facial-digital syndrome (OFD), and juvenile nephronophthisis. The severe end of the clinical spectrum is represented by the lethal disorder MKS (Barker et al., 2014). Most of the genes causative of MKS are also associated with JS, namely *CEP290, TMEM67, RPGRIP1L, CC2D2A, CEP41, MKS1, B9D1, B9D2, TMEM138, TMEM231, TCTN2, TCTN3, TMEM237, CPLANE1, CSPP1, CEP120, TMEM107*, and *TMEM216* (Parisi and Glass, 1993; Valente et al., 2010; Thomas et al., 2012; Romani et al., 2014; Bachmann-Gagescu et al., 2015; Knopp et al., 2015; Shaheen et al., 2015; Roosing et al., 2016; Slaats et al., 2016). In addition, several families with occurrence of JS and MKS in siblings have been reported (Brancati et al., 2009; Valente et al., 2010). Features of the skeletal ciliopathy JATD have been reported in several children with JS caused by mutations in *CSPP1* and *KIAA0586* (Tuz et al., 2014; Malicdan et al., 2015). Pathogenic variants in the three BBS genes *CEP290, MKS1*, and *NPHP1* have been shown to cause both BBS and JS (Leitch et al., 2008; Zaghloul and Katsanis, 2009; Knopp et al., 2015). Patients with oral-facial-digital syndrome (OFD) show features that overlap considerably with JS, as do several genes causative for OFD (Franco and Thauvin-Robinet, 2016). Patients with juvenile nephrophthisis can also show clinical overlap with JS: about 10% of individuals have extrarenal findings, including the molar tooth sign in some cases (Saunier et al., 2005). Conversely, nephronophthisis, can also be a renal manifestation in JS (Parisi and Glass, 1993). These examples illustrate the complex clinical and genetic background of JS and related ciliopathies. Preliminary genotype-phenotype correlation for some genes indicate that biallelic null alleles lead to MKS while at least one hypomorphic (e.g., missense) variant is associated with JS (Delous et al., 2007; Mougou-Zerelli et al., 2009; Tallila et al., 2009; Iannicelli et al., 2010; Romani et al., 2014). However, the molecular and cellular mechanisms that lead to a specific phenotype in patients with ciliopathies are not fully understood. Altered sonic hedgehog (SHH) signaling via defective cilia has been proposed to be the causative pathomechanism for the characteristic molar tooth sign in JS, but does not fully explain the mid-hindbrain phenotype (Spassky et al., 2008; Doherty, 2009).

Recently, mutations in *PIBF1* have been identified as a cause of JS, using a combination of a siRNA-based functional genomics screen and exome sequencing data (Wheway et al., 2015). A second publication reported a girl with a biallelic 36-bp insertion in *PIBF1* and clinical signs of JS (Hebbar et al., 2018). The patients presented with ataxia and developmental delay, ranging from mild to moderate. Imaging ranged from the classic molar tooth sign to moderate vermis hypoplasia with mildly thick superior cerebellar peduncles and characteristic superior cerebellar dysplasia (Wheway et al., 2015). In addition, thinning of corpus callosum, facial dysmorphism, hypotonia and enlarged cystic kidneys were observed in one patient (Hebbar et al., 2018). Polymicrogyria has not been described in association with *PIBF1* variants so far.

*PIBF1*, also known as *PIBF, CEP90, JBTS33*, and *C13orf24*, consists of 22 coding exons and is widely expressed in different human tissues, including the brain, kidney, and liver, with the highest expression in testis and thyroid (Fagerberg et al., 2014). *PIBF1* encodes the progesterone immunomodulatory binding factor 1 that is induced by the steroid hormone progesterone and overexpressed in highly proliferating cells (Lachmann et al., 2004; Cohen et al., 2016). The parent compound measures 90 kDa and is associated with the centrosome (Lachmann et al., 2004). A splice variant that is found in cytoplasm measures 34–36 kDa (Polgar et al., 2003; Lachmann et al., 2004). The protein regulates the immune system to maintain a normal pregnancy, may play a role in preterm labor and promotes the proliferation, migration, and invasion of astrozytoma/glioblastoma cells (Gonzalez-Arenas et al., 2014; Hudic et al., 2015, 2016; Gutierrez-Rodriguez et al., 2017). *PIBF1* encodes a centrosomal protein that may play an important role in ciliogenesis (Wheway et al., 2015). However, the precise molecular and cellular mechanisms that cause the complex JS phenotype in individuals with *PIBF1* mutations have not yet been elucidated.

Here, we report on novel *PIBF1* variants in a girl with JS. The variants were identified by whole-exome sequencing (WES) and functionally assessed in the *Xenopus* model. Our analyses demonstrate that both *PIBF1* alleles reflected loss of function variants. In general terms, the *Xenopus* model proves to be an excellent model to study the functional impact of rare genetic variants identified by diagnostic exome sequencing in patients with human ciliopathies.

## Materials and Methods

### Participants

The patient and her parents were recruited and clinically phenotyped by the Outpatient Clinic of the Institute of Human Genetics, University Hospital Heidelberg, as part of the “Genome-wide genetic analysis of rare hereditary disorders” study. Written informed consent for participation in the study and publication of study results was obtained from both parents. The study was approved by the Ethics Committee of the Faculty of Medicine at the University of Heidelberg and adhered to the tenets of the Declaration of Helsinki. A summary of the study results and its clinical implications have been published elsewhere (Evers et al., 2017). Written informed consent for the publication of this case report and parental results was obtained from the patient's parents.

### Case Report

The girl was the first child of non-consanguineous healthy parents from Germany. Her pedigree is shown in Figure 1A. She was born after 40 weeks of gestation with a birth weight of 2,620 g (1st centile), length of 48 cm (3rd centile), and head circumference (OFC) of 34 cm (20th centile). Soon after birth, spastic tetraparesis, truncal hypotonia, and feeding difficulties were noted. At age 6 months, she developed abnormal eye movements. An electroencephalogram (EEG) was normal. The girl showed a severe failure to thrive and developmental delays. Routine pediatric investigations, including basic laboratory testing and metabolic screening, resulted in normal values with the exception of mildly elevated liver enzymes (GOT: 134U/l, GTP: 164 U/l, GGT: 493 U/l), which persisted during childhood. Regular abdominal ultrasound examinations were normal with no signs of hepatic fibrosis. Ophthalmological examination including fundoscopy at age 3 years showed no abnormalities. At her first visit to the Genetic Outpatient Department at age 4 years 2 month, she presented with global developmental delay, no speech, spastic tetraplegia and a submucosal cleft palate. Her height was 85 cm (<1st centile, −4.99 SDS), her weight 11.47 kg (<1st centile, −3.08 SDS) and her OFC 49.5 cm (8th centile, −1.43 SDS). At follow up examination at age 6 years 9 months, she had a height of 98.0 cm (<1st centile, −5.49 SDS), a weight of 13.6 kg (<1st centile, −4.30 SDS) and an OFC of 50 cm (2nd centile, −2.09 SDS). cMRI at age 6 months revealed bilateral polymicrogyria in the parietal and temporal areas. Follow up MRIs at age 23 months and 2 years and 6 months showed polymicrogyria, hypoplasia of vermis cerebelli, and a mild molar tooth sign (Figures 1E–H). Chromosomal analysis and molecular karyotyping (array analysis) gave normal results. Gene panel diagnostics for Joubert syndrome by next generation sequencing of 129 known and potentially ciliopathy genes showed no pathogenic mutation. The gene panel did not include *PIBF1*, which was not known to cause JS at the time of analysis. A single gene test by Sanger sequencing of *GPR5*, a gene associated with polymicrogyria, gave normal results.

### Exome Sequencing

Genomic DNA was isolated from leukocytes of the patient and both parents by standard procedures (Miller et al., 1988). Whole exome sequencing (WES) and analysis of the sequence data of the patient and her parents was performed at the German Cancer Research Center (DKFZ) in Heidelberg, Germany, as described previously (Paramasivam et al., 2018). Variants with a minor allele frequency (MAF) >1% in the 1000 genome phase III and Exome Aggregation Consortium (ExAC) database (Lek et al., 2016) were considered common alleles and discarded, as were variants detected in 328 WES and 177 whole genome sequencing (WGS) local control samples with a frequency above 2%. Gene-based annotations from Gencode V19 were added using ANNOVAR (Wang et al., 2010). All single nucleotide variants (SNVs) and indels affecting protein sequences and variants within ±2 bases around the intron-exon junction were considered as functional. Variants were further assessed by the seven different variant effect prediction tools SIFT, PolyPhen2, LRT, MutationTaster, MutationAssessor, FATHMM, and PROVEAN from dbNSFP (Ng and Henikoff, 2003; Chun and Fay, 2009; Adzhubei et al., 2010; Schwarz et al., 2010; Reva et al., 2011; Choi et al., 2012; Liu et al., 2013; Shihab et al., 2013) and CADD scores (Kircher et al., 2014). Variants were classified according to standards and guidelines of the American College of Medical Genetics and Genomics (ACMG) (Richards et al., 2015). To confirm WES data by Sanger sequencing, exons 11 and 12 and adjacent intron boundaries of *PIBF1* (RefSeq NM_006346.2, ensemble transcript ENST00000326291.6) were sequenced using Big Dye Terminator V1.1 cycle sequencing kit and ABI 3130xl genetic analyzer. Primer sequences and PCR conditions are available upon request.

### RT PCR, qPCR, and Sequencing

Total RNA from patients, parents, and control blood was extracted using the MasterPure RNA Purification Kit (Epicentre Biotechnologies). cDNA was synthesized using random hexamer primers and reverse transcriptase RT Maxima (Fermentas). qPCR was carried out using SybrGreen mix (Thermo Scientific). Expression levels using primer pairs for the three regions of *PIBF1* (exon 2–4, exon 10–12, exon 15–17) were normalized to *ADP-ribosylation factor 1 (ARF1)*. PCR products of patient, parents and control were sequenced by Sanger sequencing (GATC).

### Western Blot Analysis of Overexpressed Protein

To analyze the expression of overexpressed PIBF1 variants, Hek293T cells were transfected in 6-well plates with 1 μg of the corresponding plasmids using Turbofect transfection reagent (Thermo Scientific. Cells were lysed 24 h post transfection and proteins were separated by 10% SDS-PAGE. For Western blot analysis, a rabbit anti-GFP antibody (1:1,000, Adgene) and a mouse anti-PIBF1 antibody (1:500, Biozol) were used.

### Protein Structure Analysis

For analyzing putative protein domains, the following algorithms were used: NCBI conserved domain search (https://www.ncbi.nlm.nih.gov/Structure/cdd/wrpsb.cgi), InterProScan (https://www.ebi.ac.uk/interpro/search/sequence-search), WoLF PSORT (https://wolfpsort.hgc.jp), and epestfind (http://emboss.bioinformatics.nl/cgi-bin/emboss/epestfind).

### *Xenopus* Injection Experiments

Adult *Xenopus laevis* frogs were obtained from Nasco (U.S.A.; https://www.enasco.com/c/Education-Supplies/Xenopus-Frogs). *Xenopus laevis* embryos were injected at the 4-cell stage into the ventral marginal zone to target the epidermal cell lineage (Moody, 2000). Translation blocking morpholino oligomere (TBMO; 5′-CCGGGACATCTTTACACTTTACATA-3′) was injected at 4 pmol per embryo. mRNAs of *EGFP* or *PIBF1* fusion constructs were injected at a dose of 0.4 pmol per embryo. Lineage tracer Fluorescein Dextran (FD, 10,000 MW, anionic, lysine fixable) was used at 50 ng per injection. Embryos were cultured until stage 30 and subsequently processed for analyses.

### RNA *in situ* Hybridization and Immunofluorescence Staining

*Xenopus* embryos were fixed using 4% paraformaldehyde solution (for *in situ* hybridization and acetylated Tuba4a staining) or Dent's (for Pibf1 and Tjp1 staining). RNA *in situ* hybridization was performed as described previously (Belo et al., 1997) using a full length digoxigenin labeled *pibf1* probe. The following reagents were used for immunofluorescence staining: anti-acetylated Tuba4a (T6793, Sigma; 1:800), anti-Pibf1 (SAB1401526, Sigma; 1:200), and anti-Tjp1 (21773-1-AP, Proteintech Europe; 1:400).

### High-Speed Video-Microscopy

Capturing of ciliary beating required flat mounting of the specimens in a chamber constructed on a slide with tape and a cover slip. Only the most ventral cells allowed differential interference contrast microscopy of the ciliary tufts, which were recorded at 600 fps using a Hamamatsu X high speed video camera. Kymographs were generated using ImageJ (https://imagej.net/Generate_and_exploit_Kymographs).

## Results

The phenotypic features of the patient (cf. section Case Report), including developmental delay, hypotonia, polymicrogyria, vermian hypoplasia, and mild molar tooth sign (Figure 1), led to the clinical diagnosis of JS. The observed liver involvement with elevated GOT/GRP is also a typical finding of this ciliopathy. A cleft palate reported here is a rare finding in JS and demonstrates its clinical overlap with oral-facial-digital syndrome. The microcephaly of the patient is not part of the classical JS spectrum, but has been reported in a patient with a *PIBF1* missense mutation (Kodani et al., 2015).

### Exome Sequencing

Exome sequencing variants were filtered as described above and only heterozygous *de novo* variants and variants being consistent with an autosomal recessive disease model were considered. Applying these filter criteria, 10 variants remained (Table 1). These were further assessed by *in silico* predicted effects on protein function, as described above (Table 1). Subsequently, a literature search was performed to gain further information about gene function and to determine if the gene had been previously associated with intellectual disability, neurological or developmental disorders in humans. This narrowed the candidate list to *PIBF1* variants c.1453C>T; p.(Q485^*^) and c.1508A>G; p.(Y503C). The variant c.1453C>T; p.(Q485^*^) was classified as pathogenic [class 5, according to ACMG criteria; (Richards et al., 2015)]. The variant c.1508A>G; p.(Y503C) was classified as variant of unknown significance [class 3, according to (Richards et al., 2015)].

**Table 1:** *De novo* and compound heterozygous variants and prediction of their functional effects.

**Affected genes**	RefSeq transcript and variant information*	**Variant status**	*in silico* parameters **: MT/MA/SIFT/PPH2(HDIV:HVAR)//FATMM/PROVEAN/LRT
ATXN1	ENST00000436367.1:exon7:c.G630T:p.Q210H	Heterozygous *de novo*	N/N/D/-:-/T/N/-
DSPP	ENST00000399271.1:exon5:c.2001_2003del:p.667_668del	Heterozygous *de novo*	Poly/-/-/-:-/-/-/-
EPS8L2	ENST00000318562.8:exon8:c.G616T:p.A206S	Heterozygous	DC/M/T/B:B/T/N/Del
EPS8L2	ENST00000318562.8:exon13:c.1071_1072insCTG:p.T357delinsTL	Heterozygous	Poly /-/-/-:-/-/-/-
OBSCN	ENST00000366707.4:exon52:c.A5292T:p.Q1764H	Heterozygous	N/N/T/D:P/T/N/N
OBSCN	ENST00000422127.1:exon94:c.20514_20515del:p.6838_6839del	Heterozygous	DC/-/-/-:-/-/-/-
PIBF1	ENST00000326291.6:exon11:c.C1453T:p.Q485X	Heterozygous	DC/-/-/-:-/-/-/-
PIBF1	ENST00000326291.6:exon12:c.A1508G:p.Y503C	Heterozygous	DC/M/D/D:D/T/D/Del
ZFHX3	ENST00000397992.5:exon9:c.C7543T:p.R2515C	Heterozygous	DC/N/T/D:B/D/N/Del
ZFHX3	ENST00000397992.5:exon8:c.G5535T:p.Q1845H	Heterozygous	DC/L/T/D:D/T//Del

* *According to Ensembl database (http://www.ensembl.org)*.

** *Obtained by prediction tools MutationTaster (MT) (Schwarz et al., 2010), MutationAssessor (MA) (Reva et al., 2011), SIFT (Ng and Henikoff, 2003), PolyPhen2 (PPH2) HDIV and PPH2 HVAR (Adzhubei et al., 2010), FATHMM (Shihab et al., 2013), PROVEAN (Choi et al., 2012) and LRT (Chun and Fay, 2009)*.

*B, benign; D, damaging; DC, disease causing; Del, deleterious; L, predicted functional effect is low; M, predicted functional effect is medium, N, neutral; Poly, Polymorphism; ProbD, probably damaging; T, tolerated; PosD, possibly damaging*.

### Expression Analysis of *PIBF1* Variants in the Patient

**Figure 2:**
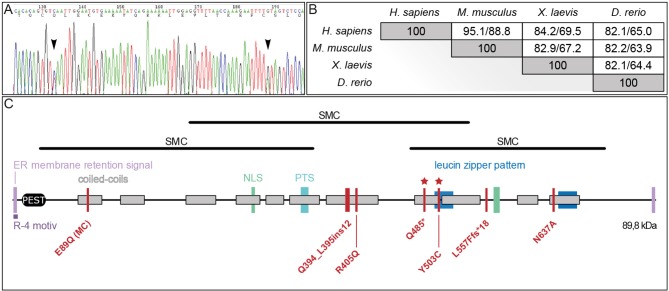
PIBF1 protein conservation and domain structure. **(A)** Sanger sequencing of *PIBF1* from RT-PCR product of the patient mRNA extracted from blood. Black arrow heads indicate position of mutations. **(B)** Conservation of amino acid sequences between human, mouse, *Xenopus*, and zebrafish PIBF1. **(C)** Putative domain structure. JS and microcephaly (MC) mutations are indicated as red bars. For details see text. *Novel mutations identified in this study.

cDNA fragments of three different regions of *PIBF1* (exons 2–4, 10–12, and 15–17) were amplified from the patient and both parents, showing a higher expression in the patient compared to mother, father, adult, and infant control (Figure S1A). Interestingly, the cDNA of exons 15–17, which are localized 3′ of the predicted premature stop codon of the variant p.(Q485^*^), showed a higher expression in the patient as well. However, whether both variants are transcribed in the patient was still unclear. Sequencing of the PCR product of the patient showed that both *PIBF1* variants could be detected in the patient (Figure 2A). The parents' cDNAs carried either the missense or the nonsense variant in heterozygous state (data not shown). This indicated that mRNA harboring the predicted pathogenic variant was not degraded by nonsense-mediated mRNA decay (NMD) and that both variants resulted in stable mRNAs. The enhanced transcription of both *PIBF1* variants in the patient could be a compensatory response to decreased PIBF1 protein levels due to protein instability of the mutants. The *PIBF1* nonsense variant p.(Q485^*^) was expected to result in the synthesis of a truncated protein lacking the C-terminal 273 amino acids. Expression analysis of wildtype (WT) and mutated EGFP-PIBF1 constructs using an anti GFP antibody showed expression of WT and both mutated proteins, the one with the missense and the one with the truncating variant at expected size (Figure S1B). This result indicated that both, missense and the nonsense variant, resulted in the synthesis of stable proteins with the nonsense variant being smaller than the WT and the missense, as expected. Analyses using an antibody against the full length PIBF1 protein could detect the WT and the missense variant, but not the truncated nonsense variant. However, the sensitivity of the antibody was far lower than that of the GFP antibody and it has not been tested on truncated PIBF1 proteins so far. Whether its epitope is in the lacking C-terminus has to be determined.

### Protein Structure and Evolutionary Conservation of *Pibf1*

A comparison of PIBF1 protein sequences from various vertebrate species revealed a high degree of conservation (Figure 2B). The *Xenopus* sequence showed 69.5% identity (84.2% similarity) to human PIBF1, very close to the mouse (Figure 2B). Functional studies at the protein level have not been reported for PIBF1 so far. Applying the NCBI conserved domain search algorithm highlighted three possible SMC-related domains (structural maintenance of chromosomes; Figure 2C). Additional searches identified a putative PEST domain, two ER membrane retention signals including one R-4 motiv, two nuclear localization sequences (NLS), a peroxisomal targeting signal (PTS), and two leucine zipper sequences as well as 11 coiled-coil domains along the 757 amino acids (Figure 2C). The three known JS mutations as well as the two novel alleles reported here localize to the C-terminal half of the protein and within coil-coiled domain, while a 6th mutation, which caused microcephaly, was found at the N-terminus, again in a coiled-coil domain (Figure 2C).

The above *in silico* analyses thus showed that the two novel mutations are located in important regions of a highly conserved JS candidate gene. In order to prove that these alleles indeed were causative for JS in the patient, they needed to be functionally tested in a relevant vertebrate model organism, particularly because the variant p.(Y503C) did not fulfill the ACMG-criteria to be classified as pathogenic or likely pathogenic (Richards et al., 2015). We chose to apply the *Xenopus* model, because of its suitability for studying ciliopathies: speed, high-throughput, and low cost of analyses (Johnston et al., 2017; Blum and Ott, 2018).

### Expression of *pibf1* in Ciliated Tissues During *Xenopus* Embryonic Development

**Figure 3:**
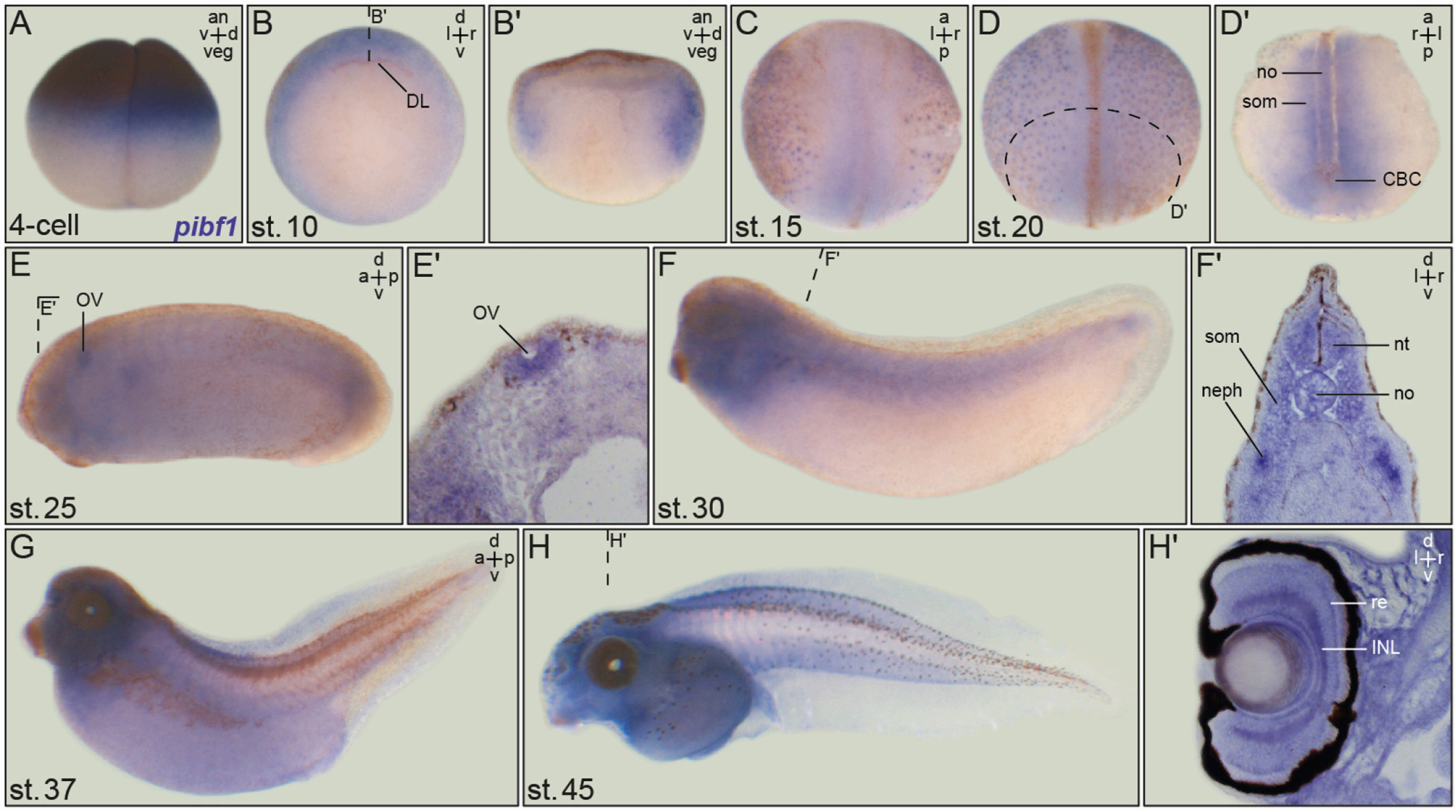
Embryonic *pibf1* expression correlates with ciliated tissues. Embryos of defined stages were analyzed for *pibf1* mRNA by whole-mount *in situ* hybridization using a digoxigenin-labeled antisense probe. **(A)** Maternal transcripts in the animal hemisphere of the 4-cell embryo. **(B)** Expression in the involuting marginal zone tissue of the early gastrula embryo. **(C)** Staining of *pibf1* in the axial and paraxial mesoderm at early neurula stages. **(D)** *Pibf1* signals in skin MCCs and axial/paraxial mesoderm. **(E)** Expression in the otic vesicle at stage 25. **(F–H)** Expression pattern in tadpoles, including the head region, somites, and nephrostomes. Histological section of the eye **(H′)** revealed signals in the retina as well as the inner nuclear cell layer. Planes of histological sections in **(B′,D′,E′,F′,H′)** are indicated in the respective panels. an, animal; a, anterior; d, dorsal; DL, dorsal lip; INL, inner nuclear layer; l, left; neph, nephrostomes; no, notochord; nt, neural tube; ov, otic vesicle; p, posterior; r, right; re, retina; som, somite; v, ventral; veg, vegetal.

As a prerequisite to functionally analyzing the putative JS alleles, we analyzed whether the endogenous *pibf1* mRNA was expressed in tissues related to cilia. Transcription of *pibf1* in embryos of defined developmental stages was analyzed using whole-mount *in situ* hybridization. Maternally deposited *pibf1* mRNA was present in the cytoplasm of the animal hemisphere in cleavage stage embryos (Figure 3A). At the onset of gastrulation, signals were found in future mesodermal tissues (Figure 3B; involuting marginal zone). From neurulation onwards, expression was found in the axial (notochord) and paraxial (somites) mesoderm (Figures 3C,D). Additionally, a dotted epidermal pattern was obvious which resembled the distribution of multiciliated cells (MCCs) in the larval skin and was maintained until the end of neurulation (Figures 3C,D). In the 2-day larva (stage 25, Figure 3E), mRNA transcripts were seen in the ciliated otic vesicle (Figure 3E′) as well as in the forming ciliated nephrostomes of the embryonic kidney. The latter staining intensified and was prominently visible in sections of stage 30 tadpoles (Figures 3F,F′). A persistent staining in the head region became more pronounced from stage 30 onwards (Figures 3F–H). Enrichment of *pibf1* transcripts in the head region as well as in the spinal cord was in agreement with the expected function of Pibf1 during neural development (Figures 3E–G). At these stages, the *pibf1* signal became less discrete, appeared more diffuse and was present at low levels in most tissues (Figures 3E–G). Histological sectioning revealed enrichment in the retina and inner nuclear cell layer (Figure 3H′). In summary, *pibf1* was expressed in many tissues harboring cilia, namely otic vesicle, nephrostomes, brain, retina, and possibly MCCs.

### Pibf1 Protein Localization in Larval Skin MCCs

**Figure 4:**
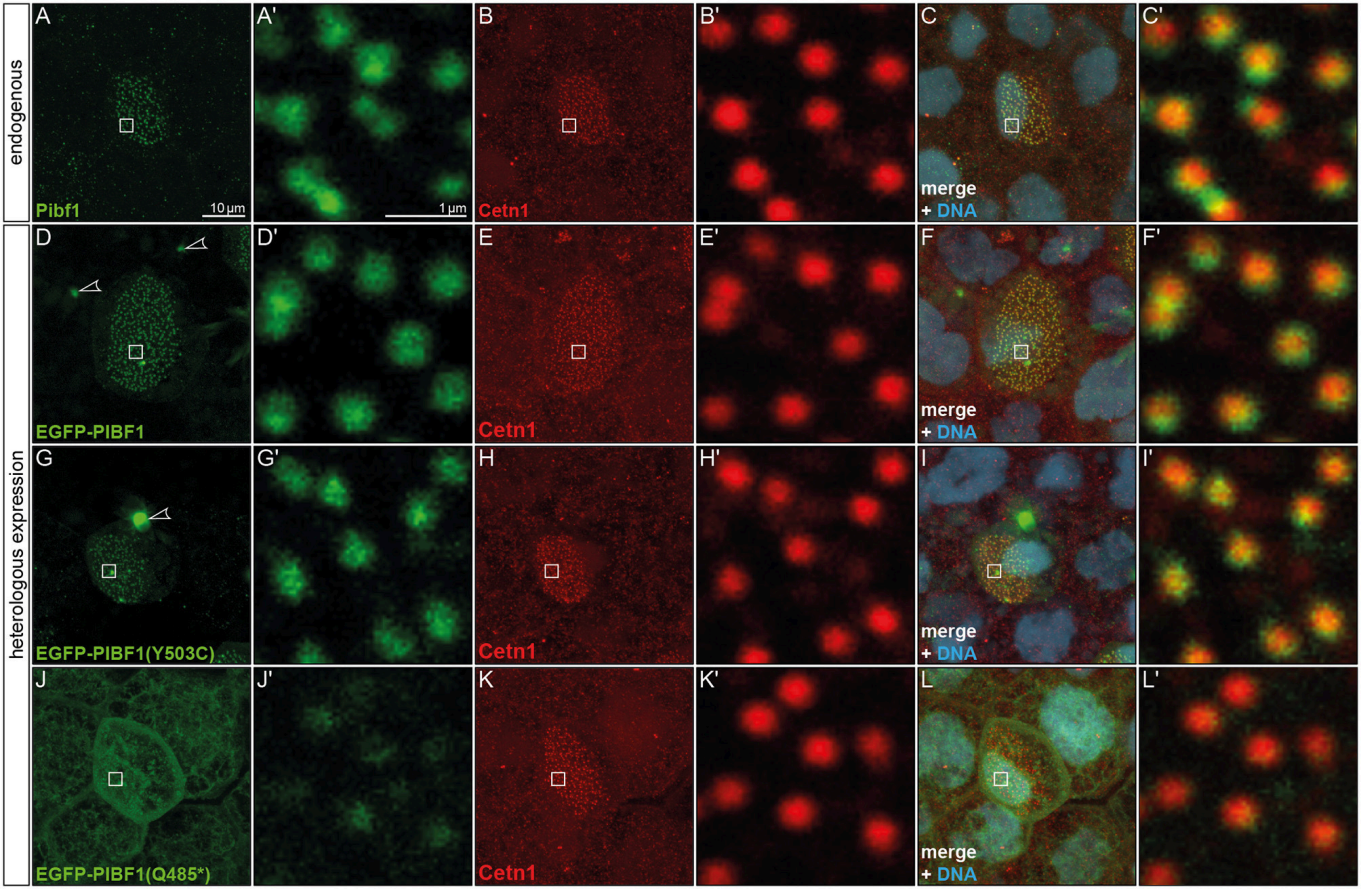
Pibf1 localization to MCC basal bodies is attenuated in nonsense JS variant. **(A–C)** Localization of the endogenous Pibf1 protein, using an anti-PIBF1 antibody **(A)** and co-staining with Centrin 1 **(B)** and Hoechst 33342 to highlight the nucleus **(C)**. **(D–F)** An EGFP-PIBF1 fusion protein recapitulates the staining of the endogenous protein. **(G–I)** Unaltered expression of the missense allele Y503C. **(J–L)** Attenuated basal body localization of the truncated Q485* variant of PIBF1. Arrowheads mark protein aggregates observed upon overexpression of EGFP fusion proteins.

JS is associated with dysfunctional primary, i.e., immotile and sensory cilia. The unexpected mRNA expression in the larval skin hinted at localization in MCCs, which harbor hundreds of motile cilia. In order to investigate the possible expression of Pibf1 in MCCs, immunofluorescence staining using a monoclonal mouse PIBF1 antibody raised against the human protein was applied. This antibody detected hundreds of spots on individual cells dotted on the larval skin (Figures 4A,A′). Co-staining with the basal body marker (Cetn1; Park et al., 2008) unequivocally demonstrated that Pibf1 indeed localized to the base of individual cilia on MCCs, specifically to basal bodies, as Pibf1 and Cetn1 partially overlapped (Figures 4A–C). Therefore, Pibf1 seemed to be a *bona fide* component of all basal bodies in ciliated cells in *Xenopus*.

In order to ascertain whether or not the missense or nonsense mutations have impact on the localization of PIBF1 to basal bodies, we cloned fusion constructs in which the N-terminus of the human WT or mutant ORFs of *PIBF1* were linked to *EGFP*. Injection of the WT PIBF1 construct into the *Xenopus* epidermis recapitulated the endogenous distribution at the basal bodies of MCCs (Figures 4D–F), demonstrating that ectopic expression of a fusion protein did not interfere with correct localization of the protein. In many cases, aggregates of fusion protein were additionally found in targeted cells (Figures 4D,G). The missense construct fully phenocopied this localization (Figures 4G–I). In contrast, the signal of the nonsense variant was much attenuated at basal bodies and was additionally found in a non-localized manner throughout the cell (Figures 4J–L). Taken together, these analyses showed that (1) Pibf1 was unexpectedly expressed in motile cilia of the larval skin; (2) Pibf1 localized to basal bodies, in agreement with its centrosomal expression in other contexts (Kim and Rhee, 2011; Kim et al., 2012); (3) the nonsense allele was much reduced in its localization to ciliary basal bodies, indicative of a ciliary function; (4) the missense allele appeared unaffected in its ciliary localization, raising questions as to the underlying mechanism of JS in the patient.

### Functional Analysis of Wildtype and Mutant *PIBF1* Alleles in the *Xenopus* Larval Skin

**Figure 5:**
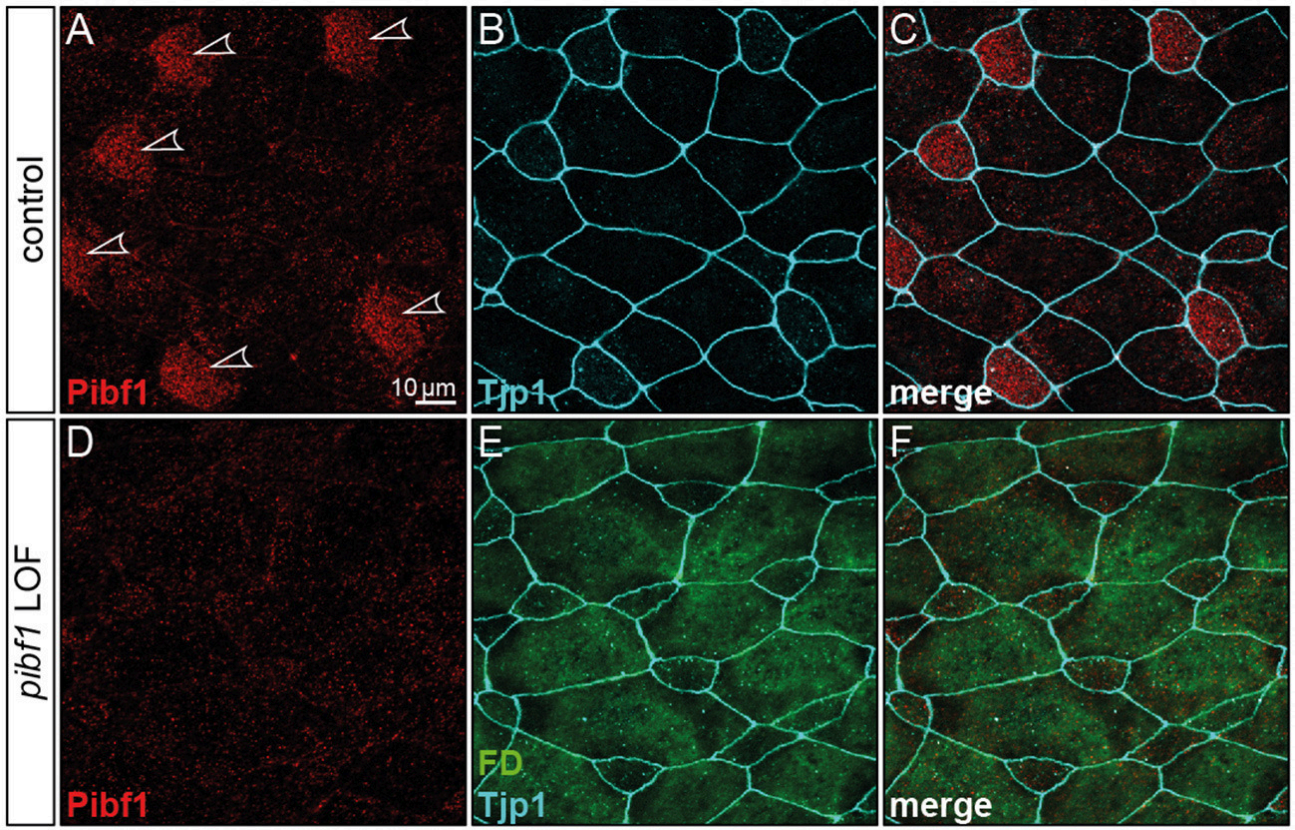
Loss of Pibf1 protein in *pibf1* morphant *Xenopus* skin MCCs. Basal body staining of Pibf1 **(A–C)** was lost in TBMO injected specimen **(D–F)**. Tjp1 immunofluorescence was used to mark cell boundaries **(B,C,E,F)**. Fluorescent dextrane (green) was co-injected as lineage tracer to control targeting of injections **(E,F)**. Arrowheads highlight MCC cilia in WT embryo.

**Figure 6:**
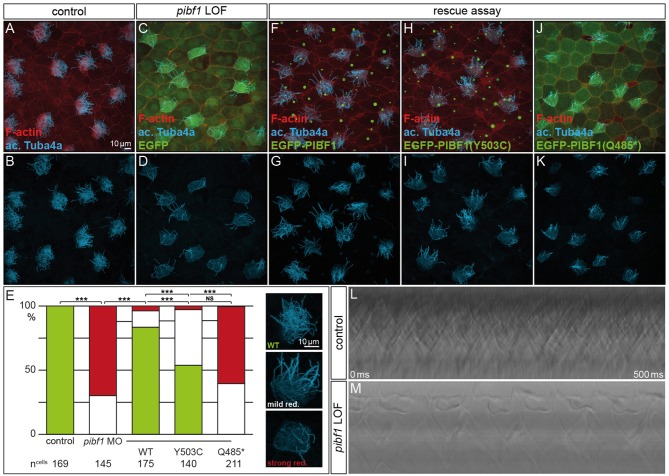
Rescue of morphant MCC ciliation is lost with mutant *PIBF1* alleles. **(A–E)** MCC ciliation in WT embryos was lost in *pibf1* TBMO-injected specimens. **(E–G)** Rescue of ciliation upon co-injection of TBMO and *EGFP-PIBF1*. **(E,H,I)** Attenuated rescue in embryos co-injected with missense *PIBF1* allele. **(E,J,K)** Defective rescue upon co-injection of the nonsense *PIBF1* allele. **(E)** Quantification of results. **(L,M)** Kymographs representing ciliary beating in WT and TBMO-injected stage 30 larval skin MCCs. ***Very highly significant, *p* < 0.01.

Although JS is not related to motile cilia, the expression and localization of Pibf1 in basal bodies of MCCs afforded the opportunity of testing whether this protein played a role in motile cilia as well and whether the mutant alleles were affected in this function. Skin MCCs of *Xenopus* larvae function in much the same way as human airway epithelia, namely in mucociliary clearance as a first line of defense against pathogens (Dubaissi and Papalopulu, 2011; Walentek et al., 2014; Blum and Ott, 2018). MCC cilia beat in a coordinated manner to move mucus, produced by goblet cells, from anterior to posterior (head to tail), and to thereby remove environmental particles and pathogens caught by the mucus layer (Brooks and Wallingford, 2014). In order to assess a possible role of *pibf1* in this process, an antisense morpholino oligomer (MO) targeting the translational start site of the mRNA (translation blocking MO, TBMO) was designed. In retinal epithelial cells (RPE-1) and inner medullary collecting duct cells (IMCD3), loss of Pibf1 resulted in fewer or absent cilia (Kim et al., 2012; Wheway et al., 2015). We therefore analyzed the presence of MCC cilia in morphant larvae that were injected with TBMO into the skin lineage at the 4-cell stage. Successful gene knockdown was proven by immunofluorescence staining for Pibf1, which demonstrated the efficient depletion of the protein from MCCs of morphant specimens (Figure 5). Immunofluorescence staining of cilia in morphant specimens was performed by staining the ciliary axoneme with an antibody against acetylated tubulin (Tuba4a). This analysis clearly demonstrated markedly reduced numbers of cilia on morphant MCCs (Figures 6A–E). Functional consequences of reduced cilia numbers were assessed by high-speed video microscopy of larval skin at stage 30. Movie S1 shows that coordinated ciliary beating was lost in morphants as compared to WT specimens. The loss of cilia was also apparent in kympgraphs from high-speed movies (Figure 6L,M). Ciliary tufts on WT and morphant MCCs were grouped into three classes, representing normal, mild, or strong reduction of cilia numbers. No unaffected ciliary tufts were retained in morphant MCCs, as displayed in Figure 6M.

Next, we asked whether heterologous expression of a WT human *PIBF1* construct was able to rescue cilia numbers in morphants. To that end, TBMO and WT PIBF1 were co-injected into 4-cell embryos and targeted to the larval skin. As shown in Figures 6E–G, the WT human gene rescued cilia numbers in a highly significant manner. In a last set of experiments, we analyzed the rescue capacity of equimolar amounts of the two novel *Pibf1* alleles identified in our JS patient. While the nonsense mutant was unable to rescue the gene knockdown (Figures 6E,H,I), a residual and attenuated rescue capability was observed when the missense mutant allele was co-injected (Figures 6E,J,K). In summary, our functional analysis of mutant *Pibf1* alleles demonstrated a role of Pibf1 in motile cilia of larval skin MCCs in *Xenopus*, and identified both nonsense and missense allele as non-functional, in agreement with the manifested JS in the patient girl.

## Discussion

Mutant gene alleles identified in patient DNAs represent a valuable resource for studying protein function and are a prerequisite for the elucidation of pathomechanisms at the molecular level. A given mutation may not, however, reveal its pathogenicity at first glance. In the case of the compound heterozygous JS patient analyzed here, one of the mutations, the truncation variant p.Q485^*^, was highlighted as pathogenic according to ACMG criteria (Richards et al., 2015). Our analysis in ciliated *Xenopus* cells confirmed its predicted pathogenicity. The disturbed localization of this variant at basal bodies in combination with the inability to rescue the ciliation phenotype of Pibf1 deficient frog MCCs identifies it as an amorphic allele. Clinically even more important, our studies of the missense-variant p.Y503C indicated a pathogenic effect of this variant, too. A careful cell by cell analysis of mutant MCCs revealed that this mutant showed an about 50% rescue capacity, significantly below the >80% achieved with the WT allele (Figure 6). It should be noted, that this analysis was performed in a tissue that is not relevant in JS patients and on motile cilia, while JS is caused by defects of primary, immotile cilia. Therefore, in the context of a *bona fide* JS target tissues, the combination of these mutations may give rise to even more pronounced defects at high frequency. Recapitulating the patient gene setup in any animal model would be experimentally more challenging, require more time and be more expensive. The attenuated rescue ability, however, unequivocally demonstrates that the missense variant is hypomorphic in nature, leading to a re-classification of the allele from “unkown significant” to “likely pathogenic,” according to ACMG criteria (Richards et al., 2015). The finding of a hypomorphic allele due to a missense mutation in combination with a null allele due to a truncating mutation is a typical finding in patients with JS and has been reported for a number of other causative genes (such as *RPGRIP1L, TMEM67, CCD2D2A*, and *TCTN3*), whereas biallelic null alleles in these genes are associated with a more severe phenotype (Delous et al., 2007; Mougou-Zerelli et al., 2009; Tallila et al., 2009; Iannicelli et al., 2010; Romani et al., 2014).

The precise role of PIBF1 in the context of ciliary biogenesis is not well-understood. All JS-associated mutations in *PIBF1* identified so far cluster in the C-terminal region of the protein [Figure 2; (Wheway et al., 2015; Hebbar et al., 2018)]. In contrast, a homozygous missense mutation that is linked to microcephaly is present within the N-terminus [Figure 2; (Kodani et al., 2015)]. The large structural maintenance of chromosomes (SMC) domains constitute almost 90% of the protein, suggesting that PIBF serves as a scaffolding factor which may dimerize with ciliary SMC proteins such as SMC1A or SMC3 (Khanna et al., 2005). A search of the protein interaction database IntAct (https://www.ebi.ac.uk/intact/) revealed a number of SMC proteins that were shown to interact with PIBF1, for example CEP63 and PCM1 (Kim et al., 2012; Gupta et al., 2015; Yachie et al., 2016). As PCM1 is not relevant for cilia formation in multiciliated mouse tracheal epithelial cells, it is not a promising candidate to explain the loss of cilia in PIBF1 depleted MCCs (Vladar and Stearns, 2007). CEP63, in contrast, is required during the centriolar duplication cycle, acts in parallel with its paralog deup1 in basal body formation in MCCs and harbors an SMC related domain (Zhao et al., 2013). The reduced number of cilia on morphant MCCs may result from such a mechanism. *Xenopus* MCC offer themselves for in-depth analyses of potential interaction partners, which are beyond the scope of the present study.

The advent of high-throughput sequencing technology, in particular whole exome sequencing (WES), has led to a revolution of genetic diagnostics of rare diseases, e.g., developmental disorders. Before the era of WES many patients had undergone a long and frustrating “diagnostic odyssey” to obtain an accurate diagnosis. This has been widely overcome with the introduction of WES, which has emerged as an effective diagnostic tool leading to diagnostic rates of around 40% in patients with previously undiagnosed neurodevelopmental or pediatric neurologic disorders [for review see (Wright et al., 2018)]. A diagnosis is essential for an optimal clinical management of the particular patient, e.g., initiation of a specific therapy or surveillance program, and appropriate access to education, social care and patient support groups (Boycott et al., 2017). A molecular diagnosis is also important for the patients' parents and other family members, in particular for informed decision-making with regard to family planning, and possibly prenatal diagnosis (PD) or preimplantation genetic diagnosis (PGD). In the case reported here, a molecular diagnosis could not have been established without the *Xenopus* analysis of the *PIBF1* missense variant and the resulting re-classification as likely pathogenic. Important consequence for the parents, who previously had decided against further children for fear of another disabled child, was that they now opt for a further pregnancy with PD.

Furthermore, identifying the molecular genetic cause of a disease is essential for a better understanding of its pathogenesis and the development of novel treatment strategies. However, interpretation of high-throughput sequencing data can be difficult. WES also uncovers many rare variants of which the functional impact is not known. Thus, a molecular diagnosis may be missed. Furthermore, recent studies in the field of cancer genetics and prenatal diagnosis indicate that unambiguous genetic results such as the finding of unclassified variants can lead to false treatment decisions and dissatisfaction with genomic testing (Kurian et al., 2017; Desai et al., 2018). Therefore, animal models are needed to verify or discard candidate disease alleles. Because of its genetic closeness, the mouse has been the model of choice to assess human genetic diseases. However, analyses in mice are costly and slow; in addition, the mouse is not suited for high-throughput analysis and cannot possibly keep up with the pace at which candidate variants keep being identified by WES. Therefore, additional and complementing animal models need to be promoted. Among the non-mammalian models, the zebrafish is widely used, while *Xenopus* is less well-known among clinical scientists. The frog, however, offers unique advantages particularly when investigating ciliopathies (Blum and Ott, 2018). The developing embryo presents its ciliated skin for a total of 3 days and allows easy and straightforward observation (including video microscopy) and functional analyses. Manipulations can be performed in a unilateral fashion, such that the non-manipulated side serves as an internal control. Given the inter-individual variability of phenotypes, this represents a unique advantage of the *Xenopus* model. The present analysis demonstrates that a syndrome like JS, which is caused by defects of primary, immotile cilia, can be successfully dissected in the context of motile cilia, as the expression analysis revealed a pan-ciliary presence of *pibf1* mRNA. This is likely true for most ciliopathies of primary cilia. Other organs that are easily addressed in the *Xenopus* model include the kidney (Getwan and Lienkamp, 2017) and heart (Duncan and Khokha, 2016). It seems, therefore, warranted to promote *Xenopus* among clinical scientist as a complementing model to mouse and zebrafish, in order to allow for the most efficient assessment of disease alleles.

## Author's Note

The authors dedicate this paper to the memory of the late Herbert Steinbeißer, who has been an inspiration to all of us and who has brought this group of people together.

## Author Contributions

TO conceived *Xenopus* experiments together with MB, performed and evaluated all *Xenopus* experiments, and wrote the manuscript together with MB and CE. MB conceived and evaluated experiments and wrote the paper. LK and ST performed mRNA/cDNA analysis of the patient and parents and westernblots. LK wrote sections of the manuscript. WES study coordination was done by CB and UM. NP and ASc performed WES analysis. Bioinformatic WES data analysis was done by NP, ASc, and MG, further evaluation of WES data was done by CE and UM. KH performed confirmation of WES variants by Sanger-Sequencing. ASe performed MRI images and their interpretation. Genotype–Phenotype correlation was done by CE, MG, KH, LK, and UM. All authors contributed to manuscript revision, read and approved the submitted version.

### Conflict of Interest Statement

The authors declare that the research was conducted in the absence of any commercial or financial relationships that could be construed as a potential conflict of interest.

## Supplementary Material

Movie S1 Uncoordinated beating of MCC cilia upon *pibf1* gene knockdown. Coordinated ciliary beating of MCC (left) was lost in *pibf1* morphant specimen (right). Note the reduced number of cilia on morphant cell. Time lapse movie was recorded at 600 fps and plays at 30 fps (0.05 × real time).

Figure S1 Expression anlayses of PIBF1 variants. **(A)** Relative expression levels of *PIBF1* exons analyzed by qPCR from patient, parents' and control blood. Shown are the mean ± SD of three independent RT-PCRs and subsequent qPCRs. *ARF1* expression was used for normalization. **(B)** Western blot analyses of overexpressed EGFP-PIBF1 constructs in HEK293 cells, using anti-GFP and anti-PIBF1 antibodies, respectively.
