# Supplementary figures and images for: The Frog Xenopus as a Model to Study Joubert Syndrome: The Case of a Human Patient With Compound Heterozygous Variants in PIBF1
Source: Front Physiol. 2019 Feb 25;10:134. doi: 10.3389/fphys.2019.00134 (PMC6397843; doi:10.3389/fphys.2019.00134)

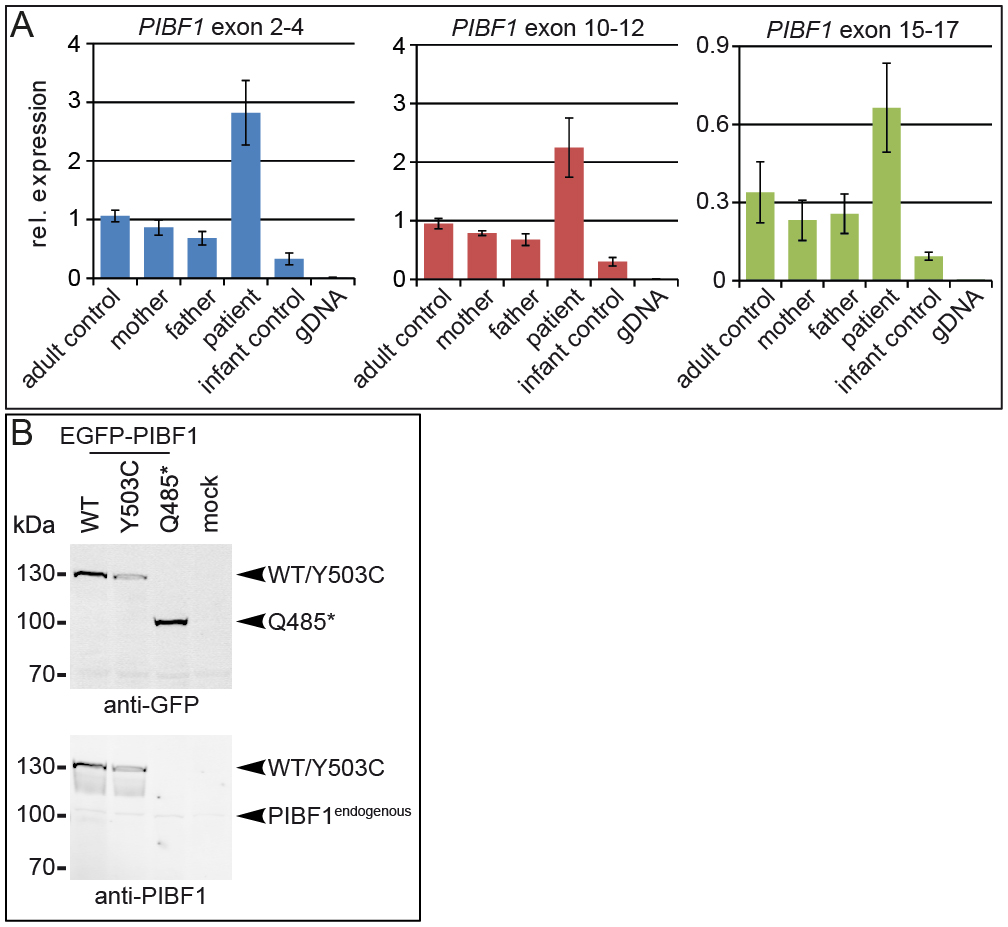

Supplement: Figure S1 — Expression anlayses of PIBF1 variants. (A) Relative expression levels of PIBF1 exons analyzed by qPCR from patient, parents' and control blood. Shown are the mean ± SD of three independent RT-PCRs and subsequent qPCRs. ARF1 expression was used for normalization. (B) Western blot analyses of overexpressed EGFP-PIBF1 constructs in HEK293 cells, using anti-GFP and anti-PIBF1 antibodies, respectively. [file Image_1.JPEG]
